# Serotonin_2C_ receptors in the nucleus accumbens are involved in enhanced alcohol-drinking behavior

**DOI:** 10.1111/j.1460-9568.2012.08037.x

**Published:** 2012-04

**Authors:** Kanji Yoshimoto, Yoshihisa Watanabe, Masaki Tanaka, Minoru Kimura

**Affiliations:** 1 Department of Forensic Medicine, Kyoto Prefectural University of MedicineKyoto 602-8566, Japan; 2 Department of Basic Geriatrics, Kyoto Prefectural University of MedicineKyoto 602-8566, Japan; 3 Department of Physiology, Kyoto Prefectural University of MedicineKyoto 602-8566, Japan; 4 Brain Science Institute, Graduate School of Brain Sciences, Tamagawa UniversityTokyo 194-8610, Japan

**Keywords:** alcohol, mesoaccumbens reward pathway, microdialysis, nucleus accumbens shell, serotonin_2C_ receptor

## Abstract

Dopamine and serotonin (5-HT) in the nucleus accumbens (ACC) and ventral tegmental area of the mesoaccumbens reward pathways have been implicated in the mechanisms underlying development of alcohol dependence. We used a C57BL/6J mouse model with increased voluntary alcohol-drinking behavior by exposing the mice to alcohol vapor for 20 consecutive days. In the alcohol-exposed mice, the expression of 5-HT_2C_ receptor mRNA increased in the ACC, caudate nucleus and putamen, dorsal raphe nucleus (DRN), hippocampus and lateral hypothalamus, while the protein level of 5-HT_2C_ receptor significantly increased in the ACC. The expression of 5-HT_7_ receptor mRNA increased in the ACC and DRN. Contents of 5-HT decreased in the ACC shell (ACC_S_) and DRN of the alcohol-exposed mice. The basal extracellular releases of dopamine (DA) and 5-HT in the ACC_S_ increased more in the alcohol-exposed mice than in alcohol-naïve mice. The magnitude of the alcohol-induced ACC_S_ DA and 5-HT release in the alcohol-exposed mice was increased compared with the control mice. Intraperitoneal (i.p.) administration or local injection into ACC_S_ of the 5-HT_2C_ receptor antagonist, SB-242084, suppressed voluntary alcohol-drinking behavior in the alcohol-exposed mice. But the i.p. administration of the 5-HT_7_ receptor antagonist, SB-258719, did not have significant effects on alcohol-drinking behavior in the alcohol-exposed mice. The effects of the 5-HT_2C_ receptor antagonist were not observed in the air-exposed control mice. These results suggest that adaptations of the 5-HT system, especially the upregulation of 5-HT_2C_ receptors in the ACC_S_, are involved in the development of enhanced voluntary alcohol-drinking behavior.

## Introduction

It is generally thought that alcohol is consumed for its positive reinforcing effects, and that chronic exposure to alcohol results in adaptations with abnormal drinking patterns (Clapp *et al.*, 2008; Koob & Volkow, 2010). The dorsal raphe nucleus (DRN) supplies the ventral tegmental area (VTA) and nucleus accumbens (ACC) with serotonin (5-HT), which modulates synaptic activity in the mesoaccumbens reward pathway (Yoshimoto & McBride, 1992; Gatto *et al.*, 1994; Koob & Le Moal, 2001). There are families of 5-HT receptors with distinct properties associated with metabotropic G-protein-coupled receptor-activated second messengers or ion channels and with amino acid sequence homologies. For example, the 5-HT_2_ family contains three receptor types, 5-HT_2A_, 5-HT_2B_ and 5-HT_2C_, that are coupled to the activation of phospholipase C (Hannon & Hoyer, 2008; Hayes & Greenshaw, 2011; Kirby *et al.*, 2011). Previously, we demonstrated that the administration of a 5-HT_1A_ receptor-selective agonist, 8-OH-DPAT, suppressed the alcohol-induced release of dopamine (DA) and 5-HT in the ACC (Yoshimoto & McBride, 1992), and that activation of the 5-HT_1A_ autoreceptor on the cell soma and dendrite decreases alcohol self-administration in drug-naïve rats (Silvestre *et al.*, 1998).

Recent studies showed that the 5-HT_2C_ receptor is linked to addictive drug effects, such as sensitization, conditioned place preference and drug self-administration (Muller & Huston, 2006; Theile *et al.*, 2009), and is associated with side-effects of drug abuse, cocaine, including anxiety, hypolocomotion and disturbances of motor function (Barnes & Sharp, 1999; Higgins & Fletcher, 2003). While 5-HT_2C_ receptor activation has been shown to decrease cocaine (Fletcher *et al.*, 2004) and alcohol (Tomkins *et al.*, 2002) self-administration, the 5-HT_2A/2C_ receptor antagonists ritanserin and ketanserin failed to alter cocaine or amphetamine self-administration (Schenk, 2000). Generally, 5-HT_2C_ receptors play an inhibitory role in the regulation of reward-related behavior (De Deurwaerdere *et al.*, 2004; Hayes & Greenshaw, 2011). However, it is not clear which 5-HT receptors are involved in synaptic functions of the mesoaccumbens reward pathway that is associated with enhanced voluntary alcohol-drinking behavior.

The goal of the present study was to understand the adaptive and compensatory processes in the mesoaccumbens reward pathway, especially the serotonergic systems, that mediate the development from occasional drinking to active and compulsive drinking that leads to excessive alcohol consumption. Using an animal model with increased voluntary alcohol-drinking behavior by exposing inbred C57BL/6J mice to alcohol vapor for 20 consecutive days, we investigated the relationship between the 5-HT_2C_ receptor in the ACC shell (ACC_S_) and the development of enhanced voluntary alcohol-drinking behavior.

## Materials and methods

The experimental designs and procedures were approved by the Committee for Animal Research, Kyoto Prefectural University of Medicine (#21-1), Kyoto, Japan, and followed the guidelines of the Ministry of Education, Culture, Sports, Science, and Technology of Japan.

### Alcohol animal models

Inbred 8-week-old male C57BL/6J mice, weighing 23–25 g (CLEA Japan, Osaka, Japan), were housed in a humidity- (60%) and temperature (23 °C)-controlled vivarium on a 12/12 h light/dark cycle (lights on 06:00 h; off 18:00 h), with *ad libitum* access to food (CE-2, CLEA Japan, Osaka, Japan) and water. The mice were handled briefly and placed in polycarbonate plastic cages (four or five per cage) for at least 1 week to acclimatize to our laboratory conditions. A 20-mL graduated glass tube was placed in each cage to record the consumption of tap water. To develop animal models for habitual or problem drinkers with lower sensitivity to alcohol, each standard mouse cage was kept in a sealed and clear plastic chamber into which alcohol vapor was intermittently administered, under the control of a timer, for 20 consecutive days in the vivarium.

Alcohol vapor was created by dripping 95% alcohol into a 2000-mL Erlenmeyer vacuum flask kept at 85 °C on a hot plate. Air was blown over the top of the flask at 1.5 L/min to vaporize the alcohol. The concentration of alcohol vapor was adjusted by varying the rate at which the alcohol was pumped into the flask, and ranged from approximately 22 to 27 mg/L (O’Dell *et al.*, 2004; Walker & Koob, 2007). The animals received alcohol vapor for a maximum of 8 h per day during the dark cycle. The alcohol vapor system was turned on at 00:00 h and off at 04:00 h for the first 5 days. For the next 5 days, it was turned on at 00:00 h and off at 06:00 h. Finally, vapor was delivered from 00:00 h to 08:00 h for the last 10 days. Control C57BL/6J naïve mice were maintained under normal conditions (air-exposed groups) throughout the experimental period.

Blood alcohol concentrations were monitored at 08:00 h in two or three mice of the experimental group during the last 5 days of the alcohol vapor exposure period to confirm the maximum concentration in the preparatory experiment. Tail blood was sampled during 6 h at the end of the alcohol vapor exposure period on the last day. A sample of 20 μL of blood was obtained by the tail bleed method from 08:00 h to 14:00 h. The blood was collected into sealed 5-mL glass vials containing 5 μL of heparin (1000 USp unit/mL) as an anticoagulant and 20 μL of *n*-propanol as an internal standard. These vials were incubated in a water bath for 20 min at 65 °C, and 0.5 mL of vapor was then injected into a gas chromatography-flame ionization detector (GC-10A, Shimadzu, Kyoto, Japan) modified according to our previous study (Yoshimoto & Komura, 1993).

### Alcohol-drinking behavior

Water was removed 12 h before performing the 4-h limited access to alcohol test. On the day of the experiment, alcohol vapor-exposed mice (*n* = 9) and control naïve mice (*n* = 8) had access to 10% (v/v) alcohol from a 20-mL graduated glass tube for a 4-h period beginning at 10:00 h. As a supplementary experiment, another group of alcohol vapor-exposed mice (*n* = 5) and control naïve mice (*n* = 5) had access to water from 20-mL graduated glass tubes for a 4-h period. The amounts consumed were measured at 0.5, 1.0, 2.0 and 4.0 h during the period of restricted access. We expressed 10% (v/v) alcohol intake as grams per kilogram per hour (the rate of alcohol consumption; g/kg/h) and total grams per kilogram in 4 hours (g/kg) and total water intake as mL per kg body weight in 4 h.

Voluntary alcohol consumption tests were performed the day after the 20-day exposure period on alcohol-exposed (*n* = 11) and alcohol-naïve control (*n* = 8) mice. Alcohol vapor-exposed mice and control mice initially had access to a 10% alcohol solution for 3 consecutive days to acclimatize them to the alcohol solution in the alcohol vapor chamber and in the normal laboratory, respectively. Then, we investigated their consumption for water or an alcohol-containing solution in the normal laboratory (Yoshimoto *et al.*, 2000). The mice had access to 10% (v/v) alcohol and tap water in graduated glass bottles (20 mL) for 10 days. The locations of the bottles were changed daily on a random basis. The daily intakes of water (mL) and 10% alcohol (mL) were measured by means of an alcohol consumption score (g/kg body weight/day) for each mouse. Body weights were recorded every second or third day. The baseline alcohol consumption of each mouse was measured individually, and consumptions of alcohol-exposed groups and control groups were compared.

### Brain monoamines and their metabolites

C57BL/6J mice of the alcohol vapor-exposed group (*n* = 10) and control naïve group (*n* = 8) were killed by decapitation at 10:00 h after alcohol inhalation for 20 consecutive days. The brain was removed, cooled on ice (this process took from 1 to 1.5 min) and then dissected using a Rodent Brain Matrix (Zivic Miller, Allison Park, PA, USA), constructed to provide coronal brain sections. Twelve brain areas, the periaqueductal gray, DRN, median raphe nucleus (MRN), VTA, substantia nigra, ACC core (ACC_C_), ACC_S_, caudate nucleus and putamen (C/P), lateral hypothalamus (LH), frontal cortex (FC), hippocampus (HP) and amygdala, were isolated using a tissue punch (1.0 mm o.d.), essentially as described by Ueda *et al.* (1998). The isolated brain tissues were rapidly frozen in liquid nitrogen and kept at −80 °C until the biochemical analysis. Each sample was weighed and homogenized using an ultrasonic-homogenizer in a mixture of 40 μL of 0.5 m perchloric acid, 30 μL of 0.1 m 2Na-EDTA and 30 μL of 3,4-dihydroxybenzylamine-HCl (DHBA) as internal standard. The homogenate was centrifuged (20 000 ***g***, 20 min), and the supernatant collected and filtered using a cellulose-acetate membrane disk (Toso, Tokyo, Japan).

The tissue levels of norepinephrine (NE), DA, 3,4-dihydroxyphenylacetic acid (DOPAC), 5-HT and 5-hydroxyindoleacetic acid (5HIAA) were determined by high-performance liquid chromatography (HPLC; 3201 Nanospace, Shiseido, Tokyo, Japan) using standard curves and adjusted for handling loss with an internal standard. Separation was performed with a reversed-phase column (TSK-Gel ODS-80Ts, 5 μm particle diameter, 150 × 2.0 mm; Toso). The mobile phase consisted of 0.1 m sodium phosphate, containing 0.1 mm EDTA-2Na with 15% methanol, 5% acetonitrile and 0.3 mm sodium-1-octane sulfonic acid, adjusted to pH 2.8 with phosphoric acid. All separations were performed at a flow rate of 0.8 mL/min. The levels of DA, 5-HT and their metabolites (DOPAC and 5HIAA) were detected with electrochemical detection (ECD; 3005 Nanospace, Shiseido), using a glassy carbon electrode set at + 0.6 V vs. an Ag/AgCl reference electrode. All values are expressed as pmol/mg. Additionally, DA and 5-HT turnover indexes, changes of which indicate the increased release of DA and 5-HT at terminals, are shown as ratios of the metabolites to their respective monoamine precursors.

### Reverse transcriptase-polymerase chain reaction (RT-PCR) analysis: RNA preparation and real-time PCR

Another group of alcohol vapor-exposed mice and control mice (*n* = 5–8), as described above, were killed by decapitation at 10:00 h at the end of the first day of exposure to alcohol vapor and after 20 consecutive days of exposure. Coronal brain sections from all mice were obtained using a 2-mm punch and quickly frozen in liquid nitrogen. Total RNA from these samples was prepared using an RNAqueous Kit (Applied Biosystems, Carlsbad, CA, USA), according to the manufacturer’s instructions. cDNAs were synthesized from 1 μg of total RNA with a Transcriptor First-Strand cDNA Synthesis Kit (Roche, Basel, Switzerland), as previously described (Tanaka *et al.*, 2009; Watanabe *et al.*, 2009). Real-time quantitative PCR was performed in a Thermal Cycler Dice Real Time System (TaKaRa Bio, Otsu, Japan) using SYBR Premix Ex Taq II (TaKaRa Bio). PCR was carried out using 40 cycles of two-step PCR as follows: 5 s at 95 °C; and 30 s at 60 °C. Primers were designed for five 5-HT receptor subtypes: *Htr1A* (5-HT_1A_); *Htr2A* (5-HT_2A_); *Htr2C* (5-HT_2C_); *Htr3* (5-HT_3_); and *Htr7* (5-HT_7_); and *DDR*_*2*_ (DA D2 receptor) and glycerol-3-phosphate dehydrogenase (Table 1). Amplification was confirmed by a melting curve to ensure the specificity of the PCR products. On the basis of analyses and calculations integrating adequate standard curves, the relative quantification of gene expression was determined.

**Table 1:** Gene names and primer sequences

Gene name	Primer sequences
*Htr1A*	5′-GGATGTTTTCCTGTCCTGGT-3′/5′-CACAAGGCCTTTCCAGAACT-3′
*Htr1B*	5′-TCACATGGCCATTTTTGACT-3′/5′-CAGTTTGTGGAACGCTTGTT-3′
*Htr2A*	5′-AGAACCCCATTCACCATAGC-3′/5′-ATCCTGTAGCCCGAAGACTG-3′
*Htr2C*	5′-ATGCCCCTGTCTCTGCTTGC-3′/5′-GCATAGCCGGTTCAATTCGC-3′
*Htr3A*	5′-CTTCCCCTTTGATGTGCAG-3′/5′-CCACTCGCCCTGATTTATG-3′
*Htr7*	5′-TTCTGCAACGTCTTCATCG-3′/5′-ATTCTGCCTCACGGGGTA-3′
*DDR*_*2*_	5′-GCAGGAAGCTCTCCCAGCAG-3′/5′-ATGATGGGGTTCACGGCACT-3′
*G3PDH*	5′-GGCATTGCTCTCAATGACAA-3′/5′-TGTGAGGGAGATGCTCAGTG-3′

*Htr1A,* 5-HT_1A_; *Htr1B,* 5-HT_1B_; *Htr2A,* 5-HT_2A_; *Htr2C,* 5-HT_2C_; *Htr3A,* 5-HT_3A_; *Htr7,* 5-HT_7_; *DDR*_*2*_, dopamine D2 receptor; *G3PDH*; glycerol-3-phosphate dehydrogenase.

### *In situ* hybridization analysis of the 5-HT_2C_ receptor

Under deep anesthesia with pentobarbital (50 mg/kg), mice were perfused via the left cardiac ventricle with 20 mL of chilled 0.1 m phosphate-buffered saline (PBS) followed by 40 mL of a fixative containing 4% paraformaldehyde in 0.1 m phosphate buffer. Brains were post-fixed in the same fixative at 4 °C overnight, and then soaked in 20% sucrose in 0.1 m PBS for 48 h at 4 °C; thereafter, frontal sections (35 μm in thickness) were cut with a cryostat and processed for *in situ* hybridization (Tanaka *et al.*, 2005). Briefly, sections were deproteinized with proteinase K and acetylated in acetic anhydride and 0.1 m triethanolamine. Sections were then incubated in a hybridization buffer containing a digoxigenin (DIG)-UTP-labeled 5-HT_2C_ riboprobe for 12 h at 60 °C. After rinsing in standard sodium citrate/50% formamide and an RNase solution, sections were transferred to a DIG coloring system (Roche Diagnostics), and reacted with 4-nitroblue tetrazolium chloride (NBT) and 5-bromo-4-chloro-3-indolyl phosphate (BCIP) at pH 8.5 for 3 h until a blue color appeared in the cytoplasm. The antisense riboprobe for mouse 5-HT_2C_ mRNA was synthesized from mouse 5-HT_2C_ cDNA (1300 bp) inserted into a plasmid vector (pGEM3; Promega, Madison, WI, USA). Transcription was carried out using 120 U of T7 RNA polymerase and 1 μg of linearized template in a reaction mixture containing a DIG-labeling mixture (Roche). A sense riboprobe corresponding to the antisense probe was synthesized, but yielded no signals on *in situ* hybridization.

### 5-HT_2C_ receptor immunohistochemistry

Coronal forebrain sections were prepared in the same manner as for the *in situ* hybridization experiments. Floating sections were processed for immunohistochemistry using antibodies against 5HT_2C_ (1 : 200, sc-15081; Santa Cruz Biotechnology, Santa Cruz, CA, USA) in PBS containing 0.1% Triton X-100 for 36 h at 4 °C. The specificity of this antibody was previously examined using fluorescence immunohistochemistry (Bubar *et al.*, 2005). For visualization using 3-3′-diaminobenzidine-4HCl (DAB; Sigma-Aldrich, St Louis, MO, USA), the sections were incubated in biotinylated anti-goat IgG (1 : 1000; Vector Lab., Burlingame, CA, USA) overnight at 4 °C and in avidin-biotin peroxidase complex solution (1 : 1000; Vector Lab) for 2 h at room temperature. Sections were exposed for 6 min at room temperature to 50 mm Tris–HCl buffer containing 0.01% DAB and 0.005% H_2_O_2_; they were then mounted on glass slides, dehydrated with a graded series of ethanol and xylene, and coverslipped with Entellan (Merk, Darmstadt, Germany). Slides were examined by light microscopy (BX50; Olympus, Tokyo, Japan).

### Western blot analysis of the 5HT_2C_ receptor in the ACC and DRN of alcohol vapor-exposed mice

Coronal sections of the ACC and DRN, obtained with a 3-mm punch, were homogenized in ice-cold PBS containing 0.5% Triton X-100 and a proteasome inhibitor cocktail (Nacalai Tesque, Kyoto, Japan). After homogenization, each sample was fractionated by centrifugation (20 000 ***g***, 15 min, 4 °C); the supernatant was recovered and subjected to a protein assay (Watanabe *et al.*, 2009). Protein extracts were separated by 12.5% sodium dodecyl sulfate–polyacrylamide gel electrophoresis and transferred onto polyvinylidene fluoride membranes. After blocking, the membranes were incubated with an anti-5HT_2C_ antibody (1 : 500) or an anti-actin antibody (1 : 1000) for 12 h at 25 °C. The membranes were then incubated with alkaline phosphatase-conjugated secondary antibodies for 1 h. Immunopositive signals were detected with the NBT and BCIP reagents.

### *In vivo* brain microdialysis

Alcohol-naïve C57BL/6J mice (*n* = 20) were lightly anesthetized with chloral hydrate and placed in a Kopf stereotaxic frame. After the skull was exposed, a burr hole was drilled for a microdialysis probe guide (CMA/7 guide cannula, CMA, Solna, Sweden) that was stereotaxically implanted using the mouse brain coordinates described by Franklin & Paxinos (1997). The microdialysis guide cannula was implanted and ended 1.0 mm above the ACC_S_ (AP, 1.2 mm anterior to the bregma; L, 0.5 mm; DV, 3.5 mm from the surface of the skull). Mice were allowed 3–5 days to recover from surgery, and were then exposed to alcohol vapor in the chamber under the same conditions as described above. After 20 consecutive days of alcohol inhalation group (*n* = 6) and control group (*n* = 6) in the normal laboratory, a mouse brain microdialysis probe (CMA/7 Microdialysis Probe, membrane length: 1.0 mm; outer diameter: 0.24 mm; steel shaft length: 7.0 mm; CMA) was inserted through the guide cannula that had been implanted over 20 days. The collections of microdialysis perfusates were then initiated approximately 3 h after inserting the probe and circulating an artificial cerebrospinal fluid (CSF) solution (in mm: NaCl, 145; KCl, 2.7; MgCl_2_, 1.0; CaCl_2_, 1.2; containing 0. mm ascorbate and pH adjusted to 7.3–7.4 with 2 mm sodium phosphate buffer) through the probe at a flow rate of 0.8 μL/min. Collections of ACC_S_ perfusates in the control naïve mice and alcohol vapor-exposed mice were carried out only after stable pretreatment baseline values for ACC_S_ DA and 5-HT were attained, which were usually after 60 min. Three baseline samples were collected every 20 min and analysed with the ECD-HPLC. Samples containing basal DA and 5-HT released into the ACC_S_ were collected into a micocentrifuge tube containing 5 μL of 0.1 N HCl and 5 μL of 0.1 m 2Na-EDTA every 20 min for a 1-h period.

### ACC_S_ alcohol perfusion

Other mice with guide cannula implanted in the ACC_S_ were maintained individually in the alcohol vapor chamber before insertion of the microdialysis probe (CMA 7 Microdialysis Probe, 1.0 mm membrane, CMA) into the ACC_S_ through the guide cannula (CMA 7 Guide Cannula, CMA). They were allowed to move freely during the course of the experiments. The collections of microdialysis perfusates were initiated over 3 h after inserting the probe and circulating an artificial CSF. The microdialysis perfusion medium was switched from normal CSF solution to one containing 100 mm alcohol for 60 min after establishing stable baseline values of DA and 5-HT release in the ACC_S_ dialysates, as compared with the control (*n* = 5) and alcohol vapor-exposed (*n* = 6) mice.

### HPLC analysis of brain microdialysis samples

Aliquots (10 μL) of microdialysis samples were assayed for DA and 5-HT using a previously described procedure (Yoshimoto *et al.*, 2000) modified for a small-bore ECD-HPLC to increase sensitivity. The mobile phase consisted of 20% (v/v) methanol, 0.1 m sodium dihydrogen phosphate, 0.2 mm SOS and 0.12 mm 2Na-EDTA (pH adjusted to 6.0 with 0.1 m disodium hydrogen phosphate). A C18 reversed-phase column (150 × 2.0 mm; Toso) was used at a flow rate of 0.8 μL/min (EP-700; EiCom, Kyoto, Japan). The ECD (ECD-700; EiCom, Kyoto, Japan) was usually set at a sensitivity of 0.5 nA/V with a potential of 0.65 V on the glassy carbon electrode. Detection limits were defined at approximately 0.05 nm for the DA and 5-HT dialysates. At the end of these experiments, each mouse was decapitated and the brain was removed. Coronal sections (40 μm) were cut using a cryostat microtome and stained with Cresyl violet to histologically verify placement of the microdialysis probe in the ACC_S_. The relative recovery rates for DA and 5-HT in these experimental conditions were about 4.4% and 3.7%, respectively.

### Effects of i.p. injection of 5-HT antagonists on voluntary alcohol-drinking behavior

After exposure to alcohol vapor for 20 consecutive days, water was removed overnight before conducting the 4-h access to alcohol test. The mice were stable and exhibited reliable behavior toward alcohol and feeding. They were given doses of 0, 0.5, 1.0 and 3.0 mg/kg of 5-HT_2C_ antagonist, SB-242084,

6-chloro-5-methyl-1-[2-(2-methylpyridyl-3-oxy)-pyrid-5-yl carbamoyl] indoline and the 5-HT_7_ receptor antagonist, SB-258719, 3-methyl-N-[(1R)-1-methyl-3-(4-methyl-1-piperidiny) propyl]-N-methylbenzesulfonamide hydrochloride (Sigma-Aldrich), i.p. SB-242084 and SB-258719 were first dissolved in deionized water with a drop of lactic acid to obtain a concentration of 0.1 mg/mL, and then further diluted to the required concentration, 0.1 μg/mL and 10 μm, with Ringer’s solution and the artificial CSF solution, respectively, just before administration. SB-242084 was injected into the alcohol vapor-exposed mice and control mice, and SB-258719 was injected into the alcohol vapor-exposed mice only. Control groups were given the same volume of vehicle. Thirty minutes after drug administration, the mice were given access to a 10% (v/v) alcohol solution. The consumption of alcohol (10% v/v) was measured at 0, 0.5, 1.0, 2.0 and 4.0 h in a normal laboratory.

### Effects of microinjection of the 5-HT_2C_ antagonist SB-242084 into the ACC_S_ on voluntary alcohol-drinking behavior

**Fig 1:**
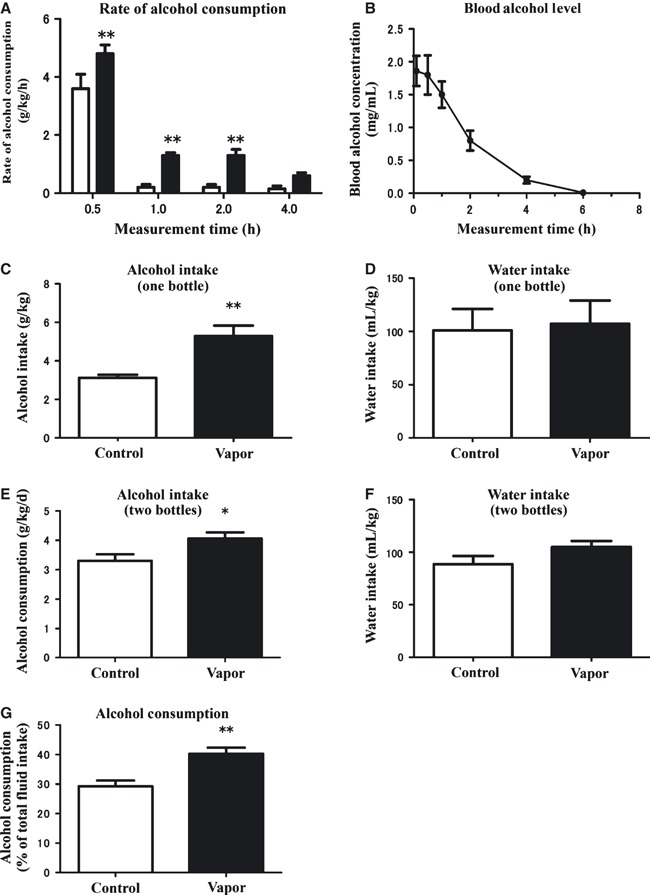
Mouse alcohol model and alcohol-drinking behavior. (A) Rate of alcohol consumption (g/kg/h) of vapor-exposed (*n* = 9) and alcohol-naïve (*n* = 8) mice during a 4-h access period. **P < 0.01 (compared with the control mice; two-way anova and then the Bonferroni *post hoc t*-test). (B) Changes of blood alcohol concentrations in C57BL/6J mice (*n* = 6) exposed to alcohol vapor for 20 consecutive days. Tail blood samples were collected at the indicated times following the end of vapor administration on the final day of alcohol inhalation. Sampling was started at 08:00 h. Values are means ± SEM. (C) Total alcohol intake (g/kg) in both groups in 4 h after a 4-h time access alcohol-drinking behavior test. **P < 0.01 (two-tailed unpaired *t*-test) as compared with the control mice. (D) Total water intake (mL/kg) of alcohol vapor-exposed (*n* = 5) and alcohol-naïve control (*n* = 5) mice in 4 h after a 4-h water access period. Values are means ± SEM. (E) Alcohol intake (g/kg/day) of alcohol vapor-exposed (*n* = 11) and alcohol-naïve control (*n* = 8) mice in the alcohol consumption test (two bottles). *P < 0.05 (two-tailed unpaired *t*-test) compared with the control mice. (F) Water intake (mL/kg/day) of alcohol vapor-exposed (*n* = 11) and alcohol-naïve control mice (*n* = 8) in the alcohol consumption test (two bottles). (G) Alcohol consumption test (% of total fluid intake) of mice exposed to alcohol vapor (*n* = 11) and alcohol-naïve control (*n* = 8) mice given the choice of water or 10% alcohol solution. **P < 0.01 (two-tailed unpaired *t*-test) as compared with the control mice.

Other alcohol-naïve C57BL/6J mice (*n* = 32) were implanted with a 24-gage microinjection guide cannula (Safelet-Cas, Nipro, Osaka, Japan) in the ACC_S_, as described above (Nakajima *et al.*, 1994). Three–5 days later they were divided into two groups, alcohol vapor exposed and control. Water was removed overnight before the 4-h limited access test to show notable alcohol-drinking behavior. No significant differences in water consumption under the influence of thirst were observed between the alcohol vapor-exposed mice and control alcohol-naïve mice (Fig. 1D). On the day of the experiment, a 27-gage injection insert was attached to a microsyringe by PE-10 tubing. The 5-HT_2C_ antagonist SB-242084 (2.5 μg/μL) or corresponding vehicle was delivered into the ACC_S_ in a final volume of 0.2 μL at a constant flow rate of 0.1 μL/min by a 10-μL syringe and a syringe pump (CMA 400; Carnegie Medicine, Sweden; control group: saline, *n* = 10 and SB, *n* = 10; vapor group: saline, *n* = 6 and SB, *n* = 6). After the microinjection, the injection cannula was left in place for an additional 2 min before removing the cannula. At 30 min after administration of the drug or vehicle, a 10% (v/v) alcohol solution was placed in each cage. The consumption of 10% (v/v) alcohol was measured at 0, 0.5, 2.0 and 4.0 h. Results were expressed as g/kg/h and g/kg/4 h.

### Statistical analyses

Data were analysed with one- and two-way repeated-measures anovas (treatment × time) using GraphPad software. *Post hoc* comparisons were carried out with the Newman–Keults test, Bonferroni *t*-test or Tukey *t*-test. In all cases, the accepted level of significance was set at *P* < 0.05 (GraphPad Prism version 5; GraphPad Software, La Jolla, CA, USA). Body weights in the alcohol inhalation and control groups were measured at 08:00 h, the starting time of each experiment.

## Results

### Alcohol inhalation model and alcohol-drinking behavior

The mean maximum blood alcohol levels in alcohol vapor-exposed mice were measured at the end of 20 consecutive days of inhalation and were approximately 1.8–2.0 mg/mL at 08:00 h during the alcohol inhalation period (Fig. 1B). The blood alcohol concentration was stable during the alcohol vapor inhalation in the preparatory experiment but decreased rapidly during the primary alcohol withdrawal stage, and was approximately 0.8 mg/mL at 10:00 h when the alcohol-drinking behavior tests were carried out. There was no significant difference in body weight between the mice exposed to alcohol vapor (*n* = 93; 24.20 ± 1.37 g) and the control mice (*n* = 56; 24.15 ± 1.30 g) in the present study.

After the vapor inhalation for 20 days, water-deprived control and alcohol-exposed mice had free access to 10% alcohol for 4 h. C57BL/6J mice exposed to alcohol vapor for 20 days showed significantly higher levels of alcohol intake at 0.5, 1.0 and 2.0 h (*F*_1,64_ = 35.08, *P* < 0.0001, and Bonferroni *post hoc* test) compared with the alcohol-naïve control group (Fig. 1A), and a significantly higher total alcohol consumption (g/kg) after 4 h (Fig. 1C). There was no significant difference in water consumption between the two groups of mice (Fig. 1D). Water deprivation had no effect on the results of alcohol-drinking behavior in the 4 h access of alcohol test. It is not likely that the alcohol vapor-exposed mice took larger amounts of the 10% (v/v) alcohol solution because they were thirstier than the control mice. Mice exposed to alcohol inhalation consumed significantly more alcohol than the control mice. Mice exposed to alcohol inhalation consumed significantly higher intake than the control mice. We then measured the amounts of alcohol consumed by mice given a choice of 10% alcohol or water in the alcohol intake test. Alcohol vapor-exposed mice consumed significantly more alcohol (4.2 g/kg/day; *P* < 0.05, unpaired Student’s *t*-test) than the control group (3.2 g/kg/day; Fig. 1E). The mice exposed to alcohol vapor also showed an increase in alcohol consumption as a percentage of total fluid intake (Fig. 1G), because there was no significant difference in water intake of the two groups of mice in the alcohol consumption test (Fig. 1F). The increase in alcohol consumption among mice exposed to alcohol vapor suggests a pharmacological action of alcohol, or alcohol adaptation, in the CNS.

### Monoamine levels in brain areas of mice exposed to alcohol vapor

To examine adaptive and compensatory processes in the mesoaccumbens reward pathway that mediate the development from occasional drinking to active and compulsive drinking that leads to excessive alcohol consumption, we investigated the effects of inhaling alcohol vapor for 20 consecutive days on the levels of DA and 5-HT and their metabolites, DOPAC and 5HIAA. We estimated the metabolism of DA and 5-HT by determining the levels of the monoamines and their metabolites and the ratio of [DOPAC] to [DA] and of [5HIAA] to [5-HT] in 12 brain areas of mice exposed to alcohol vapor (Table 2). Although the DA contents in the ACC_C_, ACC_S_ and C/P, and the 5-HT contents in DRN and ACC_S_ decreased significantly, the [5HIAA] : [5-HT] ratio increased in the DRN and ACC_S_ in mice exposed to alcohol vapor (Table 2). There were no significant changes in other areas.

**Table 2:** The levels of monoamines, DA and 5-HT, and their metabolites, DOPAC and 5HIAA

		pm/mg			Ratio	pm/mg		Ratio
Area	Group	NE	DA	DOPAC	DOPAC : DA	5-HT	5HIAA	5HIAA : 5-HT
PAG	Vapor	0.77 ± 0.21	1.10 ± 0.80	0.80 ± 0.40	0.75 ± 0.41	1.82 ± 0.61	4.63 ± 0.81	2.91 ± 1.48
	Control	0.86 ± 0.24	1.51 ± 1.00	0.80 ± 0.61	0.68 ± 0.38	2.17 ± 0.62	3.42 ± 1.07	1.90 ± 1.31
DRN	Vapor	2.14 ± 1.45	1.01 ± 0.62	1.12 ± 0.72	0.92 ± 0.63	1.57 ± 0.40^*^^*^^*^	5.01 ± 1.50	2.96 ± 1.07^*^^*^^*^
	Control	2.53 ± 1.62	1.12 ± 0.83	1.31 ± 0.84	1.12 ± 0.66	2.40 ± 0.69	3.28 ± 0.65	1.55 ± 0.99
MRN	Vapor	1.63 ± 0.43	1.15 ± 0.89	0.81 ± 0.52	0.75 ± 0.27	1.53 ± 0.51	5.24 ± 1.90	3.50 ± 1.71^*^
	Control	2.03 ± 0.69	0.93 ± 1.06	0.44 ± 0.38	0.56 ± 0.25	2.16 ± 0.75	4.00 ± 1.30	1.88 ± 0.23
VTA	Vapor	5.19 ± 1.19^*^^*^	1.78 ± 1.23	1.30 ± 0.62	0.98 ± 0.64	1.79 ± 0.77	3.41 ± 1.39	2.14 ± 1.04
	Control	3.46 ± 0.86	1.44 ± 0.97	0.97 ± 0.63	0.74 ± 0.22	2.21 ± 0.29	3.54 ± 1.28	1.58 ± 0.54
SN	Vapor	3.50 ± 0.86	0.91 ± 0.42	0.87 ± 0.30	1.21 ± 0.88	2.18 ± 0.68	3.83 ± 1.35	1.96 ± 0.99
	Control	3.85 ± 1.06	0.93 ± 0.42	1.10 ± 0.90	0.72 ± 0.38	2.77 ± 0.92	3.20 ± 0.86	1.19 ± 0.20
ACC_C_	Vapor	2.02 ± 1.40	37.84 ± 16.44^*^^*^^*^	16.07 ± 8.55	0.45 ± 0.14^*^^*^	2.11 ± 0.85	3.21 ± 1.07	1.61 ± 0.65
	Control	0.80 ± 0.19	58.34 ± 8.46	12.40 ± 2.81	0.22 ± 0.05	2.16 ± 0.41	1.90 ± 0.38	0.88 ± 0.08
ACC_S_	Vapor	3.30 ± 1.58	18.75 ± 8.50^*^^*^^*^	13.38 ± 4.42	0.85 ± 0.20^*^^*^	1.89 ± 0.54^*^^*^	3.63 ± 0.70	1.92 ± 0.49^*^
	Control	3.61 ± 2.20	26.27 ± 6.83	10.68 ± 7.11	0.41 ± 0.06	2.91 ± 0.69	2.41 ± 0.66	0.85 ± 0.25
C/P	Vapor	0.33 ± 0.29	21.01 ± 11.67^*^^*^^*^	9.55 ± 4.83	0.56 ± 0.27	1.20 ± 0.15	3.71 ± 1.13	3.46 ± 1.52
	Control	0.32 ± 0.26	29.16 ± 23.54	6.93 ± 3.67	0.35 ± 0.20	1.80 ± 0.80	2.34 ± 0.45	1.54 ± 0.57
LH	Vapor	3.07 ± 1.15	2.25 ± 0.91	1.76 ± 0.53	0.82 ± 0.20	2.56 ± 0.77	5.57 ± 1.09	2.27 ± 0.45
	Control	3.83 ± 0.26	2.05 ± 1.50	1.64 ± 0.49	1.13 ± 0.55	2.60 ± 0.79	4.72 ± 1.33	2.01 ± 0.96
FC	Vapor	1.25 ± 0.31	0.91 ± 0.41	1.01 ± 0.81	1.22 ± 0.43	0.67 ± 0.20	0.95 ± 0.31	1.45 ± 0.36
	Control	0.77 ± 0.08	1.22 ± 0.65	1.33 ± 0.78	1.42 ± 0.34	0.58 ± 0.18	0.67 ± 0.34	1.17 ± 0.60
HP	Vapor	1.11 ± 0.32	1.31 ± 0.77	1.47 ± 0.77	1.21 ± 0.77	1.33 ± 0.59	4.02 ± 1.25^*^^*^	3.50 ± 1.50^*^
	Control	0.98 ± 0.48	1.81 ± 1.01	2.01 ± 0.67	1.33 ± 0.76	1.68 ± 0.91	2.33 ± 1.22	1.50 ± 0.57
AMY	Vapor	1.48 ± 0.77	1.81 ± 0.71	2.01 ± 1.07	1.31 ± 0.71	2.11 ± 0.86	3.12 ± 0.98	1.41 ± 0.67
	Control	2.08 ± 1.01	2.31 ± 1.11	3.04 ± 1.07	1.81 ± 0.92	3.48 ± 1.01	4.11 ± 1.01	1.22 ± 0.71

5HIAA, 5-hydroxyindoleacetic acid; 5-HT, serotonin; ACC_C_, nucleus accumbens core; ACC_S_, nucleus accumbens shell; AMY, amygdala; C/P, caudate putamen; DA, dopamine; DOPAC, dihydroxyphenylacetic acid; DRN, dorsal raphe nucleus; FC, frontal cortex; HP, hippocampus; LH, lateral hypothalamus; MRN, median raphe nucleus; NE, norepinephrine; PAG, periaqueductal gray; SN, substantia nigra; VTA, ventral tegmental area. [DOPAC]/[DA] and [5HIAA]/[5-HT] ratios represent the release (turnover index) of monoamines, DA and 5-HT, respectively. Means ± SEM (vapor group, *n* = 10; control group, *n* = 8). ^*^P < 0.05, ^*^^*^P < 0.01 and ^*^^*^^*^P < 0.005 (significant difference from the drug- and alcohol-naïve C57BL/6J mice, two-tailed unpaired *t*-test).

It is important to note that the [DOPAC] to [DA] and [5HIAA] to [5-HT] ratios in the ACC_S_ and ACC_C_, and the [5HIAA] to [5-HT] ratio in the DRN significantly increased in the alcohol vapor-exposed mice. These ratios are considered an index of DA and 5-HT release (Pasinetti *et al.*, 1992). Thus, their increases indicate increases in the release of DA and 5-HT in the ACC_S_, while there was no significant difference in the levels of metabolites in the DRN and ACC_S_ after alcohol vapor inhalation (Table 2). The level of NE in the VTA, and the level of 5HIAA and ratio of [5HIAA] : [5-HT] in the MRN and HP were increased in alcohol vapor-exposed mice compared with controls (Table 2). Therefore, we propose that chronic exposure to alcohol vapor affected the DA and 5-HT systems in the ACC_S_ of the mesoaccumbens reward pathway, which may mediate increased voluntary alcohol-drinking behavior.

### mRNA expression of 5-HT and DA receptor subtypes in mice exposed to alcohol vapor

**Fig 2:**
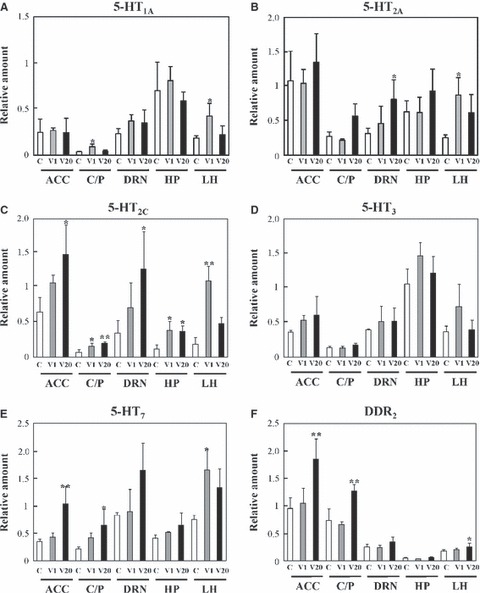
Expression of 5-HT and DA receptors subtypes. (A–F) The relative mRNA levels of (A) 5-HT_1A_, (B) 5-HT_2A_, (C) 5-HT_2C_, (D) 5-HT_3_, (E) 5-HT_7_ and (F) DDR_2_ in different brain areas of mice determined by quantitative real-time RT-PCR analysis. (C) V1, V20 denote control, alcohol vapor inhalation for 1 day, and alcohol vapor inhalation for 20 consecutive days, respectively. Values are means ± SD (*n* = 3–10) (*P < 0.05 and **P < 0.01; significant difference from alcohol- and drug-naïve control mice, anova*post hoc* Tukey test). 5-HT, serotonin; ACC, nucleus accumbens; C/P, caudate putamen (striatum); DRN, dorsal raphe nucleus; HP, hippocampus; LH, lateral hypothalamus.

The mRNA expression levels of 5-HT_1A_, 5-HT_2A_, 5-HT_2C_, 5-HT_3_, 5-HT_7_ and DDR_2_ receptors in the ACC, C/P, DRN, HP and LH were determined in mice exposed to 1 or 20 days of alcohol vapor (Fig. 2). The complete statistical analysis of the data in Fig. 2 was shown in Table 3. The expression of 5-HT_1A_ receptor mRNA in the C/P and LH was significantly increased after 1 day of exposure (Fig. 2A). The expression of 5-HT_2A_ receptor mRNA was significantly increased after 1 day in the LH and after 20 days in the DRN (Fig. 2B). 5-HT_2C_ receptor mRNA expression was significantly elevated after 1 day in C/P, HP and in all areas except LH after 20 days of exposure (Fig. 2C). No significant changes were observed for 5-HT_3_ RNA (Fig. 2D). The expression of new 5-HT_7_ mRNA was significantly increased after 1 day only in LH, and in the ACC and C/P after 20 days (Fig. 2E). Finally, the expression of DDR_2_ mRNA was significantly increased in the ACC, C/P and LH of mice exposed to alcohol vapor for 20 days (Fig. 2F). The particularly large increases in the ACC and C/P are consistent with a role of the DA system in the mesoaccumbens reward pathway. Expressions of 5-HT_2C_ mRNA in the ACC, C/P and DRN, and 5-HT_7_ mRNA in the ACC and C/P of the mesoaccumbens reward pathway increased after 20 days alcohol inhalation (Fig. 2C and E). These serotonergic receptors may play an important role in the development of voluntary alcohol-drinking behavior or alcohol dependence. The expressions of 5-HT_1A_, 5-HT_2A_, 5-HT_2C_ and 5-HT_7_ mRNA, but not 5-HT_3_ RNA, in the LH were increased only acutely after exposure to alcohol vapor for 1 day (Fig. 2A).

**Table 3:** Statistical analyses of mRNA expressions

		ANOVA		*Post hoc*
mRNA	Region	*F*-value	*P*-value	*P*-value, Tukey
5-HT_1A_	C/P	*F*_2,7_ = 7.32	0.019	V1 (*P *= 0.044)
	LH	*F*_2,9_ = 5.90	0.023	V1 (*P* = 0.035)
5-HT_2A_	DRN	*F*_2,9_ = 4.61	0.042	V20 (*P* = 0.048)
	LH	*F*_2,7_ = 4.89	0.047	V1 (*P* = 0.039)
5-HT_2C_	ACC	*F*_2,7_ = 7.32	0.019	V20 (*P* = 0.016)
	C/P	*F*_2,7_ = 10.6	0.008	V1 (*P* = 0.039)
				V20 (*P* = 0.006)
	DRN	*F*_2,9_ = 4.87	0.037	V20 (*P* = 0.036)
	HP	*F*_2,7_ = 7.07	0.021	V1 (*P* = 0.025)
				V20 (*P* = 0.042)
	LH	*F*_2,8_ = 35.1	0.0001	V1 (*P* = 0.0001)
5-HT_7_	ACC	*F*_2,7_ = 14.2	0.0035	V20 (*P *= 0.0049)
	C/P	*F*_2,8_ = 4.45	0.050	V20 (*P* = 0.043)
	LH	*F*_2,9_ = 6.91	0.015	V1 (*P* = 0.012)
	DDR_2_	ACC	*F*_2,14_ = 16.6	0.0002	V20 (*P *= 0.0003)
	C/P	*F*_2,17_ = 38.5	< 0.0001	V20 (*P* < 0.0001)
	LH	*F*_2,16_ = 3.68	0.049	V20 (*P* = 0.042)

ACC, nucleus accumbens; C/P, caudate nucleus and putamen; DRN, dorsal raphe nucleus; HP, hippocampus; LH, lateral hypothalamus.

### Histochemical analysis and Western blotting of 5-HT_2c_ in the ACC

**Fig 3:**
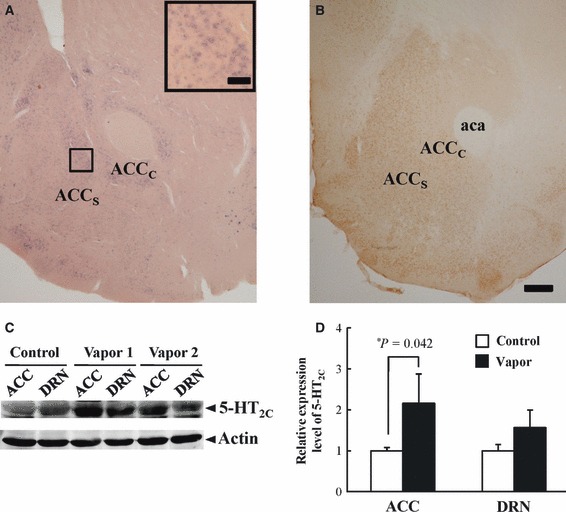
Histochemical expression and Western blotting of 5-HT_2C_. (A) *In situ* hybridization of 5-HT_2C_ mRNA in the ACC by histochemistry. The square in the upper right is a magnified picture of the transitional zone between the two regions. (B) Immunohistochemistry of 5-HT_2C_ in the ACC. The distribution of immunoreactive cells was similar to that of cells expressing 5-HT_2C_ mRNA. Scale bars: 20 μm (A); 100 μm (B). (C) Expression of 5-HT_2C_ was quantified with Western blotting analysis in different brain areas of mice exposed to alcohol vapor inhalation for 20 consecutive days, compared with the control groups. Protein extracts from alcohol vapor (lanes 3–6) or control (lanes 1 and 2) mice were subjected to Western blot analysis, followed by the immunodetection of 5-HT_2C_ (upper) and actin (lower). (D) Band densities were quantified using Image-J software. The 5-HT_2C_ band densities were normalized by the corresponding actin band densities. Values are means ± SD (*n* = 3 in each group). ^*^P = 0.042 against control, Student’s *t*-test. 5-HT, serotonin; aca, anterior commissure anterior part; ACC, nucleus accumbens; ACC_C_, nucleus accumbens core; ACC_S_, nucleus accumbens shell; DRN, dorsal raphe nucleus.

We examined the distribution of 5-HT_2C_ in the ACC of control mice by *in situ* hybridization and receptor immunohistochemistry, and analysed its expression in the ACC and DRN of alcohol vapor-exposed or control mice by Western blotting. The abundant expression of 5-HT_2C_ was observed in the ACC using *in situ* hybridization (Fig. 3A) and immunohistochemistry (Fig. 3B). 5-HT_2C_-expressing cells were distributed in the ACC_C_ and the ACC_S_.

Figure 3C shows Western blotting analysis of 5-HT_2C_ receptor and actin in ACC and DRN under control and alcohol vapor conditions. The alcohol vapor inhalation for 20 consecutive days resulted in significant increase of the 5-HT_2C_ receptor in the ACC compared with control mice (Fig. 3D).

### Brain microdialysis in the ACC_S_ of mice exposed to alcohol vapor

**Fig 4:**
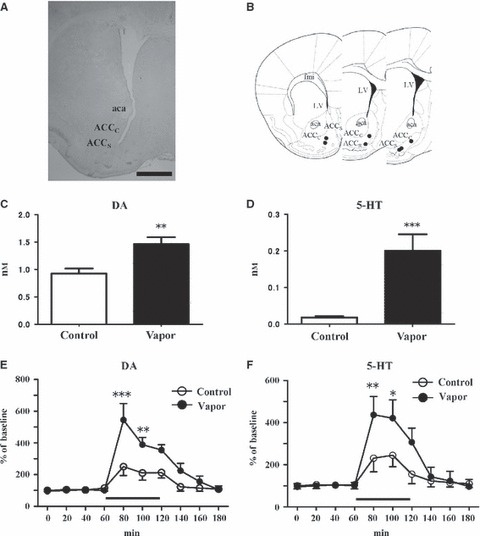
Releases of DA and 5-HT and effects of alcohol perfusion into the ACC_S_. (A) Placement of brain microdialysis guide cannulae or probes. All guide cannulae or probes were aimed at the right nucleus ACC_S_. Scale bar: 1 mm. (B) Black circles represent the guide cannula or probe tip as visualized by the manual inspection of Nissl-stained brain sections after collecting the dialysate from the ACC through the dialysis probe membrane. (C) Basal physical concentration of DA in the dialysate monitored by microdialysis in the ACC_S_ after alcohol vapor exposure for 20 consecutive days (*n* = 6). **P < 0.01 compared with the alcohol-naïve control groups (*n* = 6; two-tailed unpaired *t*-test). (D) Basal concentration of 5-HT in the dialysate monitored by microdialysis in the ACC_S_ after alcohol vapor exposure for 20 consecutive days (*n* = 5). Basal 5-HT release in the control group was at the limit of detection using our ECD-HPLC assay (*n* = 5). ***P < 0.005 comparison with the alcohol-naïve control groups (two-tailed unpaired *t*-test). (E, F) Time courses of changes in the extracellular levels of DA (E) and 5-HT (F) in the ACC_S_ of the control (open circles; *n* = 5) and alcohol vapor-exposed mice (filled circles; *n* = 6) after the perfusion of 100 mm ethanol for 60 min. After establishing a stable baseline, 100 mm alcohol was perfused with the artificial CSF for 60 min through the microdialysis probe membrane (bar). *P < 0.05, **P < 0.01 and ***P < 0.005 (compared with the control mice; one-way anova and Bonferroni’s *post hoc t*-test). Values are means ± SEM. 5-HT, serotonin; aca, anterior commissure anterior part; ACC_C_, nucleus accumbens core; ACC_S_, nucleus accumbens shell; DA, dopamine; fmi, forceps minor corpus callosum; LV, lateral ventricle.

To more closely examine the 5-HT and DA systems, we measured the release of these monoamines in the ACC_S_ via a brain microdialysis guide cannula and probe (placements are shown in Fig. 4A and B). We focused on the ACC_S_ as the target area because it is linked to alcohol-drinking behavior (Koob & Volkow, 2010) and various members of the 5-HT receptor family are expressed there (Hoyer *et al.*, 2002).

The basal extracellular concentration of DA in the dialysates of mice exposed to alcohol vapor (1.56 ± 0.25 nm) was significantly higher than that in the control mice (0.97 ± 0.15 nm; Fig. 4C). The extracellular concentration of 5-HT was also significantly higher in mice exposed to alcohol vapor (0.22 ± 0.10 nm; Fig. 4D) compared with the control group (the detectable limit of 5-HT with the ECD was approximately 1.0 pm).

### Alcohol perfusion in the ACC_S_ of mice exposed to alcohol vapor

To examine the direct effects of alcohol on monoamine release, we directly perfused the ACC with alcohol. Administration of alcohol (100 mm) through the microdialysis membrane increased the release of DA in both groups. However, the magnitude of the release was significantly greater in the alcohol vapor-exposed group (Fig. 4E; treatment: *F*_1,90_ = 20.54, *P* < 0.0001; time: *F*_9,90_ = 17.65, *P* < 0.0001). Alcohol-induced 5-HT release was also higher in the vapor-exposed mice than in the control group (Fig. 4F; treatment: *F*_1,90_ = 8.11, *P* < 0.005; time: *F*_9,90_ = 10.57, *P* < 0.0001). These results suggest that the varicosities and terminals of 5-HT neurons in the ACC_S_ of alcohol vapor-exposed mice had developed greater sensitivity to alcohol.

### Effects of i.p. injection of the systemic 5-HT antagonists on voluntary alcohol-drinking behavior

**Fig 5:**
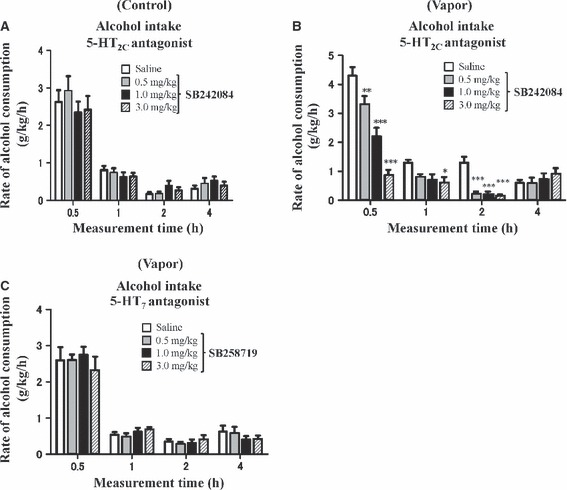
Effects of systemic 5-HT receptor antagonists on drinking behavior. (A, B) Rate of alcohol consumption (g/kg/h) over 4 h following administration of the 5HT_2C_ antagonist SB-242084 (0, 0.5, 1.0 and 3.0 mg/kg i.p.) in control mice (A) (total *n* = 44) and mice exposed to alcohol for 20 consecutive days (B) (total *n* = 32). *P < 0.05, **P < 0.01 and ***P < 0.005 compared with saline vehicle (Bonferroni *post hoc t*-test). (C) Rate of alcohol consumption (g/kg/h) over 4 h following administration of the 5-HT_7_ receptor antagonist SB-258719 (0, 0.5, 1.0 and 3.0 mg/kg i.p., total *n* = 32) in mice exposed to alcohol for 20 consecutive days. All drug treatments were administered 30 min prior to the initiation of the drinking test. Values are means ± SEM. 5-HT, serotonin.

To examine the connection between 5-HT receptors (specifically 5-HT_2C_ and 5-HT_7_ receptors) and drinking behavior, we systemically administered specific 5-HT receptor subtype antagonists and investigated alcohol-drinking behavior over a 4-h period in alcohol vapor-exposed and control mice. There was no significant effect of SB-242084 in the control mice (time × treatment: *F*_9,160_ = 1.312 n.s.; time: *F*_3,160_ = 101.8, *P* < 0.0001; treatment: *F*_3,160_ = 1.876 n.s.; Fig. 5A). On the other hand, in the alcohol-exposed mice, alcohol consumption decreased in an SB-242084 dose-dependent manner (*F*_3,116_ = 29.68, *P* < 0.0001) and altered in the time course (*F*_3,116_ = 106.7, *P* < 0.0001).Treatment with SB-242084 (0.5–3.0 mg/kg, i.p.) significantly reduced voluntary alcohol-drinking behavior in the alcohol-exposed mice (time × treatment: *F*_9,116_ = 98.62, *P* < 0.0001; Fig. 5B). We observed no significant differences in voluntary alcohol consumption in the alcohol-exposed groups following the administration of the 5-HT_7_ receptor antagonist, SB-258719 (Fig. 5C).

### Effects of microinjection of SB-242084 into the ACC_S_ on voluntary alcohol-drinking behavior

**Fig 6:**
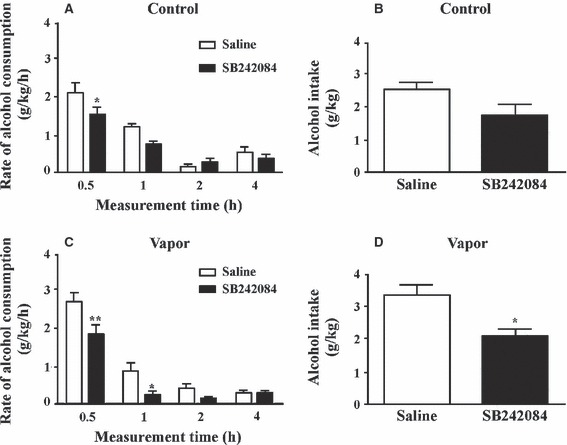
Effects of microinjection of SB-242084 into the ACC_S_ on drinking behavior. (A, C) Effects of the microinjection of 5-HT_2C_ antagonist SB-242084 into ACC_S_ of the control mice and mice exposed to alcohol vapor inhalation on the rate of alcohol consumption (g/kg/h). SB-242084 (0.5 μg/0.2 μL) or the same volume of vehicle were administered in the control (A: saline, *n* = 10; SB, *n* = 10) and alcohol vapor inhalation groups (C: saline, *n* = 6; SB, *n* = 6). Each mouse received all treatments at 30 min prior to the initiation of the test. Two-way anova and Bonferroni *post hoc* test (*P < 0.05 and **P < 0.01). (B, D) Effects of the microinjection of 5-HT_2C_ antagonist SB-242084 into the ACC_S_ on the total alcohol intake (g/kg) in the control (B: *n* = 16) and mice exposed to alcohol vapor inhalation at the end of 20 consecutive days (D: *n* = 16). *P < 0.05 (two-tailed unpaired *t*-test) as compared with the control mice. Values are means ± SEM.

We investigated the effect of microinjecting SB-242084 into the ACC_S_ on alcohol-drinking behavior over a 4-h period in the alcohol vapor-exposed and control mice. Treatment with SB-242084 (0.5 μg) significantly decreased voluntary alcohol-drinking behavior at 0.5 h (treatment: *F*_1,80_ = 6.233, *P* < 0.05; time: *F*_3,80_ = 46.27, *P* < 0.0001; and treatment × time: *F*_3,80_ = 2.206, n.s.) in the control groups, and at 0.5 and 1 h (treatment: *F*_1,48_ = 15.00, *P* < 0.005; time: *F*_3,48_ = 73.81, *P* < 0.0001; treatment × time: *F*_3,48_ = 2.932, *P* < 0.005) in the alcohol vapor-exposed mice, respectively (Fig. 6A and C). The microinjection of SB-242084 into the ACC_S_ of mice exposed to alcohol vapor inhalation caused a significant decrease in total 4-h alcohol consumption (*P* < 0.05, unpaired Student’s *t* test) compared with saline (Fig. 6D). In contrast, there was no significant difference in the control naïve mice (Fig. 6B).

## Discussion

An adequate animal model exhibiting the symptoms of alcoholism is of critical importance to understanding brain mechanisms in alcoholics. However, alcoholism is a complex behavioral disorder involving the excessive intake of alcohol, and the development of tolerance and dependence. The inhalation procedure has advantages, because the alcohol dose and duration of exposure can be precisely controlled, and the level of intoxication can be maintained stable (Becker, 2000). Chronic intermittent alcohol vapor inhalation in male C57BL/6J mice was reported to result in an increased alcohol-drinking behavior when blood alcohol concentration reached above 1.75 mg/mL (Griffin *et al.*, 2009). In the present study, C57BL/6J mice showed a significantly higher level of alcohol intake after alcohol vapor inhalation for 20 consecutive days (Fig. 1G) than after normal air inhalation (control) without any physiologically serious withdrawal syndromes or changes in body weight. C57BL/6J mice given intermittent access of 20% (v/v) alcohol on alternate days showed more than 20 g/kg/day of ethanol intake for 3 weeks, which was greater than mice given continuous alcohol access (Hwa *et al.*, 2011). In the present study the time of alcohol vapor exposure was restricted to 8 h/day for 20 days to simulate an intermittent access to alcohol solutions. An intermittent access method may best model human alcohol dependence with withdrawal symptoms. After the exposure of alcohol inhalation for 20 days, alcohol-exposed mice showed alcohol intakes of approximately 5.3 g/kg/4 h and 4.2 g/kg/day on the time access alcohol test and alcohol consumption test, respectively (Fig. 1C and E). On the other hand, C57BL/6J mice given continuous access to 10% and 20% alcohol showed about 9.3 g/kg/day and 11.8 g/kg/day of alcohol intake, respectively (Hwa *et al.*, 2011). The intermittent access to 20% alcohol drinking protocol can produce alcohol intakes of up to two to three times higher in the inbred strains of mice. Different values of alcohol consumption in the same inbred strains of C57BL/6J mice may result from different drinking metrics in the two-bottle alcohol test. The use of intermittent or restricted access to alcohol may be advantageous in the development of alcohol animal models.

The role of the 5-HT system in voluntary alcohol-drinking behavior has been extensively investigated (Lesch, 2005; Kirby *et al.*, 2011). Voluntary alcohol intakes are negatively correlated with levels of 5-HT in the mesoaccumbens reward pathway (McBride *et al.*, 1993), and low levels of 5-HT neurotransmission are associated with increased alcohol-drinking behavior (Strother *et al.*, 2005). In the selectively bred, alcohol-preferring and non-preferring lines of rats, levels of 5-HT in the FC, ACC and C/P were lower in alcohol-preferring rats (Zhou *et al.*, 1994). In the present study, lower levels of 5-HT in the DRN and an increase in the [5HIAA] to [5-HT] ratio in the DRN and ACC_S_ were found in the alcohol vapor-exposed mice that exhibited increased alcohol-drinking behavior (Table 2). An increase in the [5HIAA] to [5-HT] ratio indicates an increase in 5-HT release, as supported by its upregulation in the synaptic terminals of the ACC_S_ (Fig. 4D).

Alcohol activates the release of DA and 5-HT in the ACC, which is a key area in the mesoaccumbens reward pathway (Yoshimoto & McBride, 1992; Clapp *et al.*, 2008). Recent results indicate that the ACC_S_, but not ACC_C_, is a site that mediates the reinforcing action of alcohol, and that the ACC_S_ of alcohol-preferring rats is more sensitive to the reinforcing effects of alcohol (Engleman *et al.*, 2009). This suggests the possibility that mice with increased alcohol-drinking behavior may require alcohol to stimulate the release of sufficient amounts of DA and 5-HT in the ACC_S_ (Fig. 4C and D) as a reward. In other words, these mice consume alcohol to maintain a moderately high blood alcohol level (Fig. 1B) to adapt to or compensate for the increased activity of 5-HT in the DRN-ACC_S_ 5-HT system.

Our regimen of alcohol vapor inhalation resulted in increased 5-HT release in the ACC_S_ and decreased 5-HT content in the DRN and ACC_S_ (Fig. 4D; Table 2), and induced an elevation of 5-HT availabilities in the synaptic clefts of the ACC_S_. Furthermore, a higher release of 5-HT of the ACC_S_ with alcohol perfusion via a microdialysis membrane in mice exposed to alcohol vapor inhalation than in control mice (Fig. 4F) would estimate more activated serotonergic neurons in the ACC_S_. These results may be related to inhibition of clearance independent of the serotonin transporter (SERT), because physiologically relevant doses of alcohol inhibited the clearance of 5-HT from extracellular fluid in the HP of a knockout mouse with the genetic inactivation of SERT (Boyce-Rustay *et al.*, 2006; Daws *et al.*, 2006). Increased voluntary alcohol drinking to sustain the biological availability of 5-HT in the ACC_S_ is associated with the adaptation of 5-HT_2C_ receptor functions as evidenced by increased 5-HT_2C_ expression in the ACC (Figs 1G, 2C and 3C). Together, these observations suggest that alterations in 5-HT_2C_ receptor functions and 5-HT transmission in the ACC_S_ contribute to the drive to consume alcohol.

In the development of enhanced voluntary alcohol-drinking behavior in mice exposed to alcohol vapor we found the increased expression of specific 5-HT and DA receptor subtypes in the reward pathway (Fig. 2). The expression of 5-HT_2C_ receptor in mice exposed to alcohol vapor was significantly elevated in the ACC of the mesoaccumbens reward pathway (Fig. 3D), suggesting that enhanced alcohol drinking in the alcohol models was strongly attributed to adapted 5-HT_2C_ receptor functions in the ACC. 5-HT_2A_ mRNA in the DRN was also increased following alcohol inhalation (Fig. 2B). Stimulation of 5-HT_2A_ and 5-HT_2C_ receptors coupled to the Gq protein activates phospholipase C (Muller & Huston, 2006); thus, activation of the phosphatidylinositide-signaling pathway may be involved with the development of increased alcohol drinking. And it follows then, that increased 5-HT_2C_ receptor density in the alcohol models might have caused the upregulation of serotonergic function in the ACC_S_ participating in the development of voluntary alcohol-drinking behavior. Furthermore, the expression of 5-HT_7_ receptor mRNA, coupled with Gs proteins (Barnes & Sharp, 1999), in mice exposed to alcohol vapor was increased in the ACC and C/P (Fig. 2E). In the present study, no changes in 5-HT_3_ receptors were observed in the development of enhanced alcohol-drinking behavior (Fig. 2D).

Both the i.p. administration and microinjection into the ACC_S_ of SB-242084 suppressed the consumption of alcohol in a dose-dependent manner (Figs 5B and 6C). When the 5-HT_2C_ antagonist was administered during the primary stage of alcohol withdrawal, alcohol drinking was suppressed (Figs 5B and 6C). It was suggested that the alcohol-exposed mice showed the alteration of 5-HT_2C_ receptor sensitivity to alcohol with the increase of density in the ACC (Fig. 3D). In accordance with this, the 5-HT_2C_ receptor antagonist mianserin blocked the development of alcohol sensitization when given in the acute or primary phase of daily alcohol injection (Ferraz & Boerngen-Lacerda, 2008). Similarly, pretreatment with 1 mg/kg SB-242084 subcutaneously completely prevented cocaine-seeking behavior (Burbassi & Cervo, 2008). We explain this as follows: the 5-HT_2C_ antagonist SB-242084 antagonized 5-HT_2C_ receptor function and may alter the clearance of 5-HT in the ACC_S_ of alcohol vapor-exposed mice, thereby suppressing alcohol-drinking behavior (Figs 5B and 6D). Regarding the relationship between the activation of 5-HT clearance and alcohol-drinking behavior, it was reported that pharmacological agents, fluoxetine, imipramine and amitriptyline, which inhibit 5-HT reuptake from the synapses, reduce alcohol-drinking behavior (Nestler & Aghajanian, 1997). Furthermore, SERT knockout mice exhibit decreased alcohol preference and consumption (Boyce-Rustay *et al.*, 2006). We observed increases in 5-HT_2C_ receptor density after chronic intermittent alcohol vapor inhalation that contribute to the increase of 5-HT release in the ACC_S_ (Figs 2C and 4D). During chronic alcohol vapor exposure, there are adaptive changes related to tolerance to alcohol, or increased alcohol-drinking behavior, that result in increases in 5-HT_2C_ receptor density that contribute to the excitatory effects and the increase of 5-HT release in the ACC_S_ (Figs 2C and 4D). The potentiation of selective serotonin reuptake inhibitor (SSRI) fluoxetine-induced elevation in hippocampal 5-HT levels was reproduced by the 5-HT_2C_ receptor-selective antagonist SB-242084. 5-HT_2C_ receptor antagonist augmented the action of SSRI as the availability of synaptic 5-HT (Cremers *et al.*, 2004). It was suggested that the compound SB-242084 shows the possibility of dual properties, working as an antagonist of 5-HT_2C_ receptor as well as an indirect 5-HT uptake blocker, thereby increasing the availability of synaptic 5-HT in the ACC_S_ of mice exposed to alcohol vapor.

It was reported that 5-HT_2C_ receptor-mediated phosphoinositide hydrolysis associated with phospholipase C was elevated more in alcohol-preferring P rats (alcohol-drinking models) than in non-preferring rats (Pandey *et al.*, 1996; Qu *et al.*, 2005). This suggests that the local injection of SB-242084 antagonizes the 5-HT_2C_-receptor-mediated phosphoinositide hydrolysis in the ACC_S_. Thus, both an elevation of Ca^2+^ with the increase of inositol 2,4,5-triphosphate and the activation of 5-HT_2C_ receptor-mediated phosphoinositide hydrolysis associated with phospholipase C in the ACC_S_ appear to be involved in Ca^2+^-triggered serotonergic neural firing mechanism (Sudhof, 2004) and the enhanced alcohol-drinking behavior in mice exposed to alcohol vapor.

In addition to the 5-HT_2C_ receptor, other 5-HT receptors may also be involved in increased alcohol consumption. Although the expression of 5-HT_7_ mRNA also increased in mice exposed to alcohol vapor (Fig. 2E), the 5-HT_7_ receptor antagonist had no significant effect on alcohol-drinking behavior in mice exposed to alcohol vapor (Fig. 5C). There is accumulating evidence that the 5-HT_7_ receptor plays a role in psychiatric disorders (Bonaventure *et al.*, 2002; Mnie-Filali *et al.*, 2007; Sarkisyan *et al.*, 2010). Interestingly, the administration of alcohol vapor for just 1 day resulted in an acute, but transient, increase in the expression of 5-HT_1A_, 5-HT_2A_ and 5-HT_2C_, and 5-HT_7_ receptor mRNAs in the LH (Fig. 2).

In conclusion, we found that 8-week-old male C57BL/6J mice exhibited enhanced alcohol-drinking behavior after being exposed to alcohol vapor for 20 consecutive days. There was no significant difference in water intake and body weight between the alcohol vapor-exposed mice and control mice. This occurred in association with an increased expression of the 5-HT_2C_ receptors in the ACC_S_. The administration of a 5-HT_2C_ antagonist, not only through systemic infusion but also through local injection into the ACC_S_, suppressed the increase in alcohol-drinking behavior and alcohol preference in alcohol vapor-exposed mice. These effects were not observed after the administration of a 5-HT_7_ antagonist. Therefore, our findings support the hypothesis that the activation of 5-HT_2C_ receptors in the ACC_S_ of the mesoaccumbens reward pathway plays a pivotal role in increased alcohol-drinking behavior.

## Abbreviations

5HIAA: 5-hydroxyindoleacetic acid
5-HT: serotonin
ACC: nucleus accumbens
ACC_C_: nucleus accumbens core
ACC_S_: nucleus accumbens shell
BCIP: 5-bromo-4-chloro-3-indolyl phosphate
C/P: caudate nucleus and putamen
CSF: cerebrospinal fluid
DA: dopamine
DAB: 3-3′-diaminobenzidine-4HCl
DIG: digoxigenin
DOPAC: 3,4-dihydroxyphenylacetic acid
DRN: dorsal raphe nucleus
ECD: electrochemical detection
FC: frontal cortex
HP: hippocampus
HPLC: high-performance liquid chromatography
LH: lateral hypothalamus
MRN: median raphe nucleus
NBT: 4-nitroblue tetrazolium chloride
NE: norepinephrine
PBS: phosphate-buffered saline
RT-PCR: reverse transcriptase-polymerase chain reaction
SERT: serotonin transporter
SSRI: selective serotonin reuptake inhibitor
VTA: ventral tegmental area
